# Longer-term (≥ 2 years) survival in patients with glioblastoma in population-based studies pre- and post-2005: a systematic review and meta-analysis

**DOI:** 10.1038/s41598-020-68011-4

**Published:** 2020-07-15

**Authors:** Michael T. C. Poon, Cathie L. M. Sudlow, Jonine D. Figueroa, Paul M. Brennan

**Affiliations:** 1 Usher Institute, University of Edinburgh, Edinburgh, UK; 2 Brain Tumour Centre of Excellence, Cancer Research UK Edinburgh Centre, University of Edinburgh, Edinburgh, UK; 3 Centre for Clinical Brain Sciences, University of Edinburgh, Chancellor’s Building, Edinburgh BioQuarter, 49 Little France Crescent, Edinburgh, EH16 4SB UK

**Keywords:** Cancer epidemiology, CNS cancer, Epidemiology, Outcomes research

## Abstract

Translation of survival benefits observed in glioblastoma clinical trials to populations and to longer-term survival remains uncertain. We aimed to assess if ≥ 2-year survival has changed in relation to the trial of radiotherapy plus concomitant and adjuvant temozolomide published in 2005. We searched MEDLINE and Embase for population-based studies with ≥ 50 patients published after 2002 reporting survival at ≥ 2 years following glioblastoma diagnosis. Primary endpoints were survival at 2-, 3- and 5-years stratified by recruitment period. We meta-analysed survival estimates using a random effects model stratified according to whether recruitment ended before 2005 (earlier) or started during or after 2005 (later). PROSPERO registration number CRD42019130035. Twenty-three populations from 63 potentially eligible studies contributed to the meta-analyses. Pooled 2-year overall survival estimates for the earlier and later study periods were 9% (95% confidence interval [CI] 6–12%; n/N = 1,488/17,507) and 18% (95% CI 14–22%; n/N = 5,670/32,390), respectively. Similarly, pooled 3-year survival estimates increased from 4% (95% CI 2–6%; n/N = 325/10,556) to 11% (95% CI 9–14%; n/N = 1900/16,397). One study with a within-population comparison showed similar improvement in survival among the older population. Pooled 5-year survival estimates were 3% (95% CI 1–5%; n/N = 401/14,919) and 4% (95% CI 2–5%; n/N = 1,291/28,748) for the earlier and later periods, respectively. Meta-analyses of real-world data suggested a doubling of 2- and 3-year survival in glioblastoma patients since 2005. However, 5-year survival remains poor with no apparent improvement. Detailed clinically annotated population-based data and further molecular characterization of longer-term survivors may explain the unchanged survival beyond 5 years.

## Introduction

Glioblastoma multiforme is the most common primary malignant brain tumour in adults with an incidence rate of 3.7 per 100,000 person-years, though geographical variation exists^1^. Despite an increasing understanding of the underlying pathophysiology, glioblastoma remains an incurable disease with high mortality^2^. A landmark clinical trial in 2005 demonstrated that the addition of concomitant and adjuvant temozolomide to radiotherapy provided an additional survival benefit to patients diagnosed with glioblastoma^3^. Multiple clinical trials had investigated novel therapies that showed promise in pre-clinical and early phase studies, but to date there have been no major additions to the treatment armamentarium for newly diagnosed patients since 2005. The median survival in the intervention arm of the 2005 trial was 14.6 months^3^, but there is uncertainty about whether survival benefit from clinical trials is translated to the population^4,5^. Clinical trial participation itself is associated with better survival^6^, which may be caused by the preferential inclusion and exclusion criteria into clinical trials. In clinical practice not all patients are eligible for the trial standard of care involving maximal surgical debulking, chemotherapy and radiotherapy, for example because of co-morbidities, poor functional status or tumour location within the brain. There is also a paucity of data on long-term GBM survivors.

We systematically reviewed population-based studies that reported overall survival after glioblastoma to characterize survival beyond 2 years and investigate whether survival has changed since the landmark 2005 trial.

## Methods

### Protocol and registration

We registered this systematic review in PROSPERO (CRD42019130035) and reported it in accordance with PRISMA and MOOSE guidelines.

### Eligibility criteria

We included all population-based studies reporting overall survival at ≥ 2 years in adults with glioblastoma. Studies reporting overall survival in all types of brain tumor from which data could be extracted for glioblastoma were also eligible. There was no language restriction. We excluded conference abstracts, studies without data on overall survival at ≥ 2 years, studies with less than 50 participants, and studies published before 2003 because contemporary imaging facilities and provision, which impact on time of diagnosis and patient management, were generally not available during their recruitment periods.

### Information sources and search

We searched MEDLINE and Embase on 15 March 2019 using a combination of search terms for glioma or glioblastoma, population-based, registry, and survival or mortality (Supplementary material Appendix 1). We screened bibliographies of eligible studies for any studies missed by the electronic searches.

### Study selection

After removing duplicate records, two reviewers (MTCP and PMB) screened all titles and abstracts. Studies passing the initial screening underwent full eligibility assessment against the inclusion and exclusion criteria. We resolved any disagreements or uncertainties through discussion between the authors.

### Data collection process

We developed a data collection tool using a subset of eligible studies. One reviewer (MTCP) extracted data from eligible studies against the data collection tool. A second reviewer (PMB or JDF) resolved uncertainties by discussion.

### Data items

We collected data on study characteristics including year of publication, country of population, registry or database used, recruitment period, any study-specific selection criteria, total sample size, median age, gender, number and proportion of patients receiving different treatments (surgical resection, chemotherapy, radiotherapy), and number and proportion of patients surviving at 2, 3, 5, and 10 years. As cancer registries generally do not routinely collect the specific chemotherapy drug and its regimen, we could not quantify the proportion of patients receiving multimodal therapy. If a study reported survival data stratified by recruitment period, we extracted the stratified survival data. Therefore, a study may contribute to meta-analyses of more than one recruitment period. When studies only reported survival data on a graph, we used Plot Digitizer (https://plotdigitizer.sourceforge.net/) to extract data.

### Risk of bias assessment

As there is no published risk of bias tool specifically for evaluating population-based observational studies without a pre-defined exposure, we assessed risk of bias based on the preliminary ROBINS-E tool developed by GRADE^7, 8^. The assessment aimed to evaluate the risk of bias affecting the survival estimate in the study. Categories assessed for bias were patient selection, diagnostic certainty, handling of missing data, and outcome measurement (Supplementary Table S1). Based on risk of bias assessment in each category, we rated the overall risk of bias as low, moderate, serious, or critical.

### Summary measures

Summary measures were proportions of patients with glioblastoma surviving at 2, 3, 5, and 10 years.

### Synthesis of results

We stratified studies according to whether the recruitment period was before 2005 or during or after 2005 as a proxy to wider use of multimodal treatment in the later period, informed by the 2005 landmark trial. This dichotomy does not imply a definite use of multimodal treatment since not all patients received this treatment and clinical practice of offering this treatment varies. If several publications described the same population, we included the largest sample size. Our target population was all individuals diagnosed with glioblastoma, therefore we excluded studies with selected cohorts—for example, additional exclusion criteria based on age or treatment limit—from the meta-analyses. We meta-analysed survival data at 2, 3, and 5 years from population-based studies stratified by recruitment period using Stata 16.0 (StataCorp) using a random-effects model. We quantified heterogeneity using the I^2^ statistic.

### Additional analyses

By analysing non-overlapping patients stratified by recruitment period, we inevitably excluded some studies from the meta-analyses. Additional analyses of these excluded studies with recruitment period starting before and ending after 2005 allowed us to compare their pooled estimates with the primary analyses. Sensitivity analyses restricting to low risk of bias studies assessed how this affected the meta-analyses.

We observed that survival estimates from East Asian studies appeared higher than European and North American studies. To assess the contribution of the East Asian studies to the heterogeneity observed, we performed sensitivity analyses by meta-analysing the same survival data without studies of East Asian populations.

Further sensitivity analyses included moving the proxy year to 2006 to represent the time-point after which multimodal treatment was likely to be more common. We also meta-analysed studies where patient recruitment started before and ended after 2005. These studies contained some patients also included within populations contributed to the main analyses. However, no patients were included more than once in this additional meta-analysis.

## Results

### Study selection and characteristics

**Figure 1:**
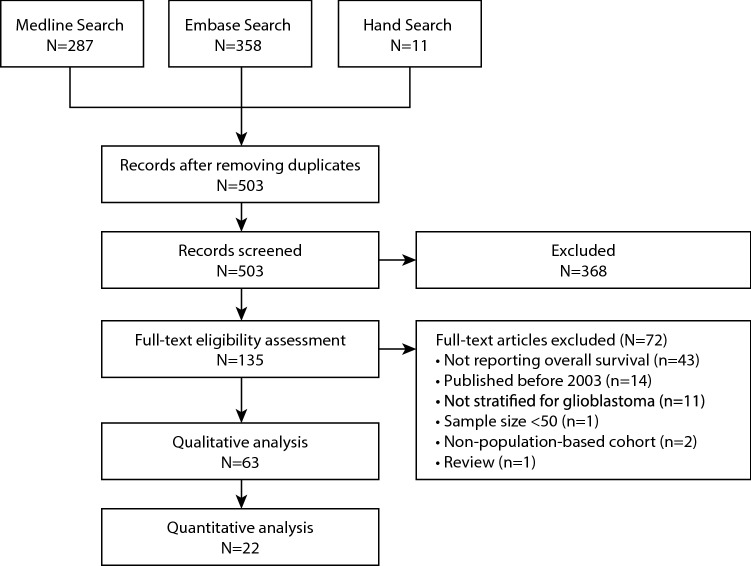
PRISMA flowchart.

Our search strategy retrieved 503 records. There were 135 potentially eligible studies, of which 63 were included in this review after full eligibility assessment (Fig. 1)^9–37^. One study reported two distinct populations^14^. Therefore, there were 63 eligible studies representing 64 populations from 17 countries that reported overall survival at ≥ 2 year in patients with glioblastoma. Detailed study characteristics are presented in Supplementary Table S2 (p 4–7). The United States was the most frequently reported population (n = 29; 45%). Twenty-seven studies reported survival estimates in Western European populations (including Switzerland and the United Kingdom), and three in Canada. Of the remaining studies, four were from East Asian populations and one from Australia. The median length of recruitment period was 8.5 years (IQR 5–14 years). Median age ranged from 58 to 75 years. In studies that did not use the predominantly male Veterans Health Administration (VA) database, the percentage of females ranged from 32 to 61%. The percentage of patients receiving each of the following treatments ranged from 25 to 100% for resective surgery; 39–100% for radiotherapy; 7–100% for chemotherapy. There were 55 (87%) studies reporting 2-year survival, 31 (49%) reporting 3-year survival, 32 (51%) reporting 5-year survival, and 3 (5%) reporting 10-year survival.

### Risk of bias

Based on our risk of bias assessment, 29 (46%) studies had low risk of bias (Supplementary Table S2). Nine studies included glioblastoma cases without histological confirmation. Fourteen studies (22%) had a sample size of less than 300. Demographic information (age and gender) were provided in 30 (48%) studies. Only 21 (33%) studies reported the proportion of patients receiving each of resective surgery, radiotherapy, and chemotherapy.

### 2-year survival

**Figure 2:**
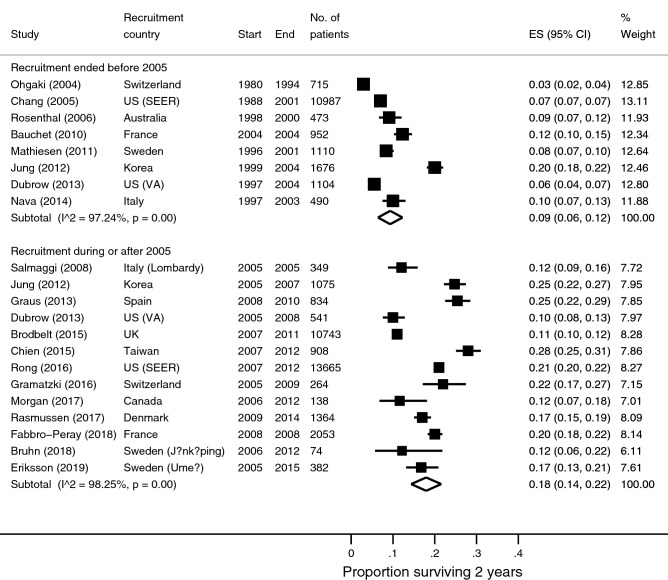
Forest plots of 2-year overall survival stratified by recruitment period.

Sixty-seven survival data points stratified by recruitment period were available from 55 studies reporting 2-year survival. There were 16 survival estimates for patients recruited before 2005, 30 for those recruited in studies that spanned across 2005, and 21 for those recruited during or after 2005. After reviewing the information sources for the sample sizes of these studies, survival data from non-overlapping patients were available in eight studies where patient recruitment ended before 2005, and 13 where recruitment started in or after 2005. Table 1 shows the characteristics of these studies. The pooled 2-year survival estimate for the earlier period was 9% (95% confidence interval [CI] 6–12%; n/N = 1,488/17,507; I^2^ = 97.2%; Fig. 2); the pooled estimate for the later period was 18% (95% CI 14–22%; n/N = 5,670/32,390; I^2^ = 98.3%; Fig. 2). Seven populations had 2-year survival available at both the earlier and later periods, enabling within-population comparisons: Surveillance, Epidemiology, and End Results (SEER) in the US^13,31^, Veterans Health Administration (VA) in the US^15^, Italy^26,35^, Switzerland^18,28^, France^9,17^, Sweden^16,24^ and Korea^22^. The survival estimates were higher during the later period compared with the earlier period in all seven populations. Three studies observed at least doubling of survival. The increase in survival ranged from 2 to 8% in the other studies.

**Table 1:** Study characteristics of 22 eligible studies included in the quantitative analyses.

Study	Country	Recruitment	Period^b^	RoB^c^	HDx	N	Age^d^	% female	% SR	% ChT	% RT	2 yr	3 yr	5 yrs
Bauchet (2010)^9^	France	2004–2004	E	1	1	952	64	38	56	59	68	●	–	–
Chang (2005)^13^	US (SEER)	1988–2001	E	3	1	10,987	64	43	74	–	74	●	–	●
Iwamoto (2008)^20^	US (SEER)	1994–2002	E	4	1	5,909	–	55	70	7	45	○	●	○
Mathiesen (2011)^24^	Sweden	1996–2001	E	1	1	1,110	–	–	–	–	–	●	–	–
Nava (2014)^26^	Italy	1997–2010	E	1	1	1,254	–	36	91	–	–	●	●	●
Ohgaki (2004)^28^	Switzerland	1980–1994	E	3	0	715	61	–	–	–	–	●	●	–
Rosenthal (2006)^32^	Australia	1998–2000	E	2	1	473	–	–	–	–	–	●	●	●
Dubrow (2013)^15^	US (VA)	1997–2008	E & L	1	1	1645	–	3	74	43	73	●	●	●
Eriksson (2019)^16^	Sweden (Umeå)	1995–2015	E & L	1	1	571	–	38	–	–	–	○	●	●
Jung (2012)^22^	Korea	1999–2007	E & L	1	1	2,751	–	–	–	–	–	●	●	●
Brandes (2014)^10^	Italy	2001–2013	L	4	1	139	59	38	–	100	100	○	●	–
Brodbelt (2015)^11^	UK	2007–2011	L	1	1	10,743	57	40	80	25	–	●	–	●
Bruhn (2018)^12^	Sweden (Jönköping)	2001–2005	L	2	1	143	–	38	34	–	–	●	–	–
Chien (2015)^14^^,a^	Taiwan	2007–2012	L	1	1	908	–	–	–	–	–	●	–	–
Fabbro-Peray (2018)^17^	France	2008–2008	L	1	1	2053	64	40	59	90	90	●	–	●
Gramatzki (2016)^18^	Switzerland	2005–2009	L	1	1	264	61	38	81	60	70	●	●	–
Graus (2013)^19^	Spain	2008–2010	L	2	1	834	62	39	66	61	72	●	●	–
Johnson (2018)^21^	US (SEER)	2006–2012	L	1	1	12,873	–	41	61	51	75	○	●	–
Morgan (2017)^25^	Canada	2006–2012	L	1	1	138	61	39	83	65	87	●	–	–
Rasmussen (2018)^30^	Denmark	2009–2014	L	1	1	1,364	64	61	–	–	–	●	●	●
Rong (2016)^31^	US (SEER)	2007–2012	L	4	0	13,665	63	42	76	–	60	●	–	●
Salmaggi (2008)^35^	Italy (Lombardy)	2005–2005	L	3	0	349	60	36	70	100	89	●	–	–

*GBM* glioblastoma multiforme, *RoB* risk of bias, *HDx* all cases with histological diagnosis, *SR* surgical resection, *ChT* chemotherapy, *RT* radiotherapy, *KPS* Karnofsky Performance Score, *TMZ* temozolomide, *NOS* not otherwise specified, *yr* years, *%* percentage, *VA* Veterans Health Administration, *SEER* Surveillance, Epidemiology and End Results Program, *OH* Ohio, *CA* California, *NC* North Carolina.

^a^This is a single study reporting survival data on two separate populations of GBM patients.

^b^This denotes the analyses in which the study contributed survival data; E = recruitment before 2005 (earlier); L = recruitment during or after 2005 (later).

^c^Risk of bias relates to survival estimate reported in the study. Scores are based on assessment of patient selection, diagnostic certainty, handling of missing data and outcome measurement. Studies are categorized into four risk of bias groups: (1) low; (2) moderate; (3) serious; (4) critical.

^d^Median or mean age of cohort in years depending on which was reported in the study.

### 3-year survival

**Figure 3:**
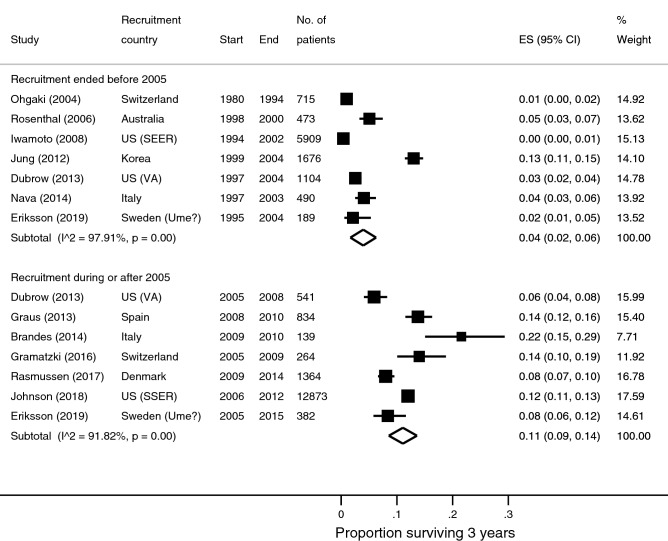
Forest plots of 3-year overall survival stratified by recruitment period.

Thirty-five survival data points were available from 31 studies reporting 3-year survival. There were nine survival estimates for patients recruited before 2005, 16 for those where recruitment spanned across 2005, and ten for those recruited during or after 2005. There were seven studies contributing to each meta-analysis for the earlier and later period (Table 1). Pooled 3-year survival was 4% (95% CI 2–6%; n/N = 325/10,556; I^2^ = 97.9%; Fig. 3) for the earlier period, and 11% (95% CI 9–14%; n/N = 1900/16,397; I^2^ = 91.8%; Fig. 3) for the later period. There was at least doubling of 3-year survival in all four populations (SEER^20,21^, VA^15^, Italy^10,26^, and Sweden^16^) that allowed a within-population comparison.

### 5-year survival

**Figure 4:**
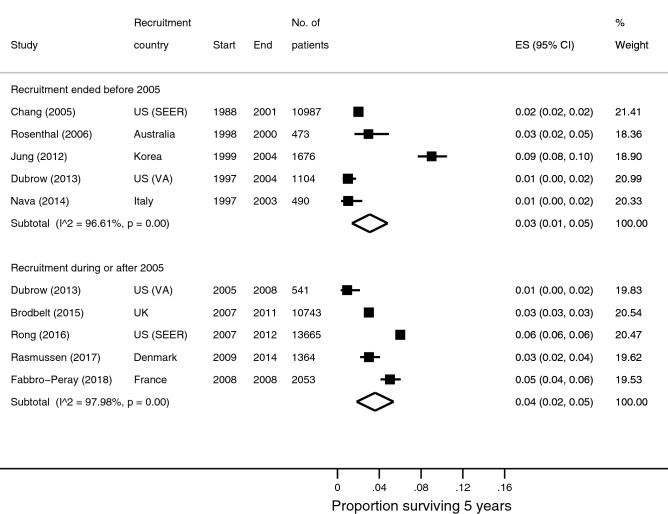
Forest plots of 5-year overall survival stratified by recruitment period.

Thirty-seven survival data points were available from 32 studies reporting 5-year survival. There were seven survival estimates for patients recruited before 2005, 22 for those recruited in studies that spanned across 2005, and seven for those recruited during or after 2005. Meta-analyses from five studies (with no duplicated patients) in each time period (Table 1) yielded pooled survival estimate of 3% (95% CI 1–5%; n/N = 401/14,919; I^2=^96.6%; Fig. 4) for the earlier period, and 4% (95% CI 2–5%; n/N = 1,291/28,748; I^2^ = 98.0%; Fig. 4) for the later period. The US VA^15^ and SEER^13,31^ populations allowed within-population comparison. Survival increased from 2^13^ to 6%^31^ in the SEER population but remained at 1% in the VA population^15^.

### 10-year survival

Only 3 (5%) studies reported 10-year survival^29,33,37^. Two studies used the United States SEER database^33,37^, while one was from Canada using a national cancer registry^37^. The recruitment periods ranged from 9 to 16 years. Each study had more than 10,000 patients. All three studies had serious or critical risk of bias for their survival estimates. Both studies from the United States included reported survival only in patients who had surgical resection, and one of them excluded those who died within 1 month of surgery. About 25% patients in the Canadian study did not have a histological diagnosis of glioblastoma. The estimate of 10-year survival ranged from 0.3^33^ to 3%^37^. No comparison in relation to 2005 was possible as all study periods started before and ended after 2005.

### Sensitivity analyses

Restricting quantitative analyses to low risk-of-bias studies showed the respective pooled survival estimates for the earlier and later periods were: 11% and 18% at 2 years; 5% and 8% at 3 years; and 3% and 3% at 5 years (Supplementary Fig. S1). Because survival estimates from East Asian populations were consistently higher than the pooled estimate, we carried out sensitivity analyses excluding the East Asian studies from meta-analyses. The respective pooled survival estimates without the East Asian populations for the earlier and later periods were: 8% and 16% at 2 years; 2% and 11% at 3 years; and 2% and 4% at 5 years (Supplementary Fig. S2). Instead of using year 2005 as a proxy time-point after which wider use of multimodal therapy occurred, we stratified studies by their recruitment period into before 2006, and during or after 2006. There was no change to the number of studies available for meta-analyses in the earlier period. In the later period, meta-analyses at 2, 3 and 5 years had 5, 3, and 1 fewer studies, respectively. The respective pooled survival estimates for the earlier and later period using 2006 as the proxy time-point were: 9% and 19% at 2 years; 4% and 12% at 3 years, and 3% and 4% at 5 years (Supplementary Fig. S3).

Meta-analyses of studies that did not contribute to the main analyses are presented in Supplementary Material (Fig. S4). These studies contain some overlapping patients with populations in the main analyses. The pooled estimates of the proportion of patients surviving at 2 and 3 years were 12% and 8%, respectively. Both were between the pooled estimates for the earlier and later periods. At 5 years, the pooled estimate was 4%.

### Older populations

Regardless of recruitment period, there were five studies that restricted their cohorts to patients ≥ 65 years^20,27,34^ and ≥ 70 years of age^23,36^. Populations in these studies were based in the United States—four from SEER^20,27,34,36^ and one from the National Cancer Database (NCDB)^23^. The 2-year survival estimates ranged from 2^20^ to 11%^23^, and 3-year survival ranged from 0.4^20,36^ to 5%^23^. The 5-year survival was 0.2% in the only study reporting at this time point^20^. One study had within-population comparison for the earlier and later recruitment periods and reported an increase in survival estimate at 2 and 3 years from 2 to 7% and from 0.9 to 3%, respectively^36^.

## Discussion

This systematic review of 63 population-based studies of which 22 were included in the primary analyses found that 2-year survival was 18%, 3-year survival was 11%, and 5-year survival was 4% in patients diagnosed with glioblastoma during or after 2005. There was a doubling of 2- and 3-year survival in contemporary populations compared with patients treated prior to 2005. These survival gains were also evident in older (≥ 65 years) patients. However, there was no apparent improvement in 5-year survival.

The most significant development in clinical management of glioblastoma in the past two decades has been the landmark trial of radiotherapy plus concomitant and adjuvant temozolomide^3^. This clinical trial among 573 patients with a median follow-up of 28 months demonstrated that when compared with radiotherapy alone, the combination therapy improved the median survival from 12.1 to 14.6 months. The reported 2-year survival was 8% in the radiotherapy alone group, and 20% in the combination therapy group. These survival probabilities are very similar to our findings for 2-year survival from the meta-analyses of earlier and later time periods. Within-population comparisons, which should be less prone to confounding, confirmed a higher survival in the later versus earlier period. Although it is not possible to directly examine whether this improvement is secondary to the use of multimodal therapy, there was a rapid increase in the use of adjuvant and concomitant temozolomide with radiotherapy after the trial publication^38^, at least in the United States. The European^39,40^ and the United States^41^ neuro-oncology guidelines have established multimodal treatment for glioblastoma as the standard of care in many countries. Hence, it is possible that the increased use of multimodal therapy contributed to the improvements observed. Our findings also demonstrate the value of using health data to assess the impact of therapeutic advances on a disease at a population level. This approach can evaluate the real-world risk–benefit ratio of treatment at scale for treatments that have significant side-effects.

International variations in incidence of malignant astrocytoma^42^ and overall survival, may suggest different aetiologies, underlying disease processes, or risk exposure such as ethnicity^1^. Investigations into these factors may elucidate different underlying pathological mechanisms. Differences in cancer registration practice could also contribute to survival variation. The International Cancer Benchmarking Partnership (ICBP), which comprises 13 jurisdictions from 6 countries, has reported differences in capturing incidence date^43^. Furthermore, the increased use of brain imaging^44^ and high prevalence of incidental findings^45^ may further contribute to the lead-time bias.

There has been significant progress in understanding genomic drivers implicated in glioma tumorigenesis. Multidimensional data from genomics studies such as The Cancer Genome Atlas (TCGA)^46^, REMBRANDT study^47^, and the Chinese Glioma Genome Atlas (CGGA)^48^ have identified molecular signatures associated with glioma subtypes and their prognoses^49^. Isocitrate dehydrogenase (IDH) mutation and 1p/19q co-deletion are most prominent markers associated with a more favourable prognosis^49^. The importance of these markers are reflected in the 2016 update of the WHO classification of central nervous system (CNS) tumours^50^, which incorporates molecular markers into the definition of tumour entities, in addition to histological features. Future studies should examine outcomes and therapy responses amongst these molecular subtypes.

Studying clinical phenotypes can gain knowledge of glioblastoma disease mechanisms. In a recent study using radiographical and transcriptomics data, it was suggested that female and male patients with glioblastoma have different molecular mechanisms^51^. Studies have suggested that longer-term survivors may have different clinical and tumour characteristics compared to shorter-term survivors. Patients with glioblastoma surviving 5 years or more are younger^52–54^ and have better performance status at the time of diagnosis^53,54^ compared with shorter-term survivors. Methylation of the O^6^-methylguanine-DNA-methyltransferase (MGMT)^55,56^ and IDH^55^ mutation are more common in patients surviving 3 years compared with those who died before 3 years. However, studies of long-term survivors are challenging given that there are very few subjects, with a varying degree of clinical detail. The observation that the proportion of patients surviving 5 years has not changed, may indicate a molecularly distinct group of patients within this heterogeneous disease. In order to clarify this hypothesis, future studies should include comprehensive clinical details with molecular markers in a large patient cohort.

This systematic review and meta-analysis presented international real-world data drawn from population-based studies. We evaluated longer-term survival beyond the median survival observed in the landmark trial, and we were able to compare survival in the era of contemporary management to an earlier period. Our data also addressed the survival uncertainty in the older population. However, there were some limitations to our study. A high proportion of eligible studies used the same national database. We adopted a strategy to prevent any patient from being included more than once in our analyses. Inevitably this led to the omission of patients recruited in some time periods. However, our sensitivity analyses comparing the pooled estimate of these omitted studies to that of the main analyses showed a confirmatory trend of survival improvement (Supplementary Fig. S4). There was considerable between-study heterogeneity despite excluding studies with a high risk of bias and examining heterogeneity in our sensitivity analyses. Unmeasured factors such as unrecorded variables, missing treatment data, and variations in data coding would contribute to the variation of survival estimates^57^. Patients in some studies did not have histological confirmation; patients with better prognosis associated with lower grade gliomas or other brain tumours may have been included. This ascertainment bias is unlikely to have significantly affected our findings since studies in the meta-analyses, where some patients did not have histological confirmation, had similar survival to other studies. While this review examines the trend in survival in relation to a landmark clinical trial, we were unable to evaluate directly the pattern of treatment and its impact on survival due to limited treatment information available from eligible studies. Within-population comparison of different time periods, which may partially control for unmeasured factors, was only possible in a few eligible studies. We are, therefore, unable to directly attribute the survival trend observed to a change in treatment patterns. We used the year of 2005 as a proxy time-point for the introduction of wider use of multimodal therapy. Although some studies specified the more prevalent use of multimodal therapy, not all would have taken up recommendations from the clinical trial. However, our sensitivity analysis showed no substantial change in trend and having the cut-off year at 2005 allowed more studies to be included (Supplementary Fig. S3). Survival improvement may be attributable to other factors in addition to the multimodal treatment, such as more prevalent use of neuroimaging leading to earlier diagnosis, strengthening of multidisciplinary neuro-oncology care, and advances in neurosurgical techniques.

## Conclusions

Overall estimates of survival among patients with glioblastoma have at least doubled since 2005 to 18% at 2 years and 11% at 3 years. This may reflect treatment response to modern therapeutic approaches. However, longer term survival remains poor and there appears to be a lack of improvement in 5-year survival. Large population-based studies with detailed clinical characteristics would clarify whether there has been an increase in the use of multimodal therapy and whether multimodal therapy affects survival beyond 5 years. Differences in msolecular markers of tumour subtypes may shed light on the underlying disease mechanisms that influence survival.

## Supplementary information

Supplementary file1.

Supplementary file2.

Supplementary file3.
