# Supplementary material for: Longer-term (≥ 2 years) survival in patients with glioblastoma in population-based studies pre- and post-2005: a systematic review and meta-analysis
Source: Sci Rep. 2020 Jul 15;10:11622. doi: 10.1038/s41598-020-68011-4 (PMC7363854; doi:10.1038/s41598-020-68011-4)
Supplement: Supplementary file 3 — Supplementary file3. [file 41598_2020_68011_MOESM3_ESM.docx]

**Supplementary material**

Contents

[Appendix 1. Search Strategy 2](#_Toc15890449)

[Supplementary Table S1. Risk of bias assessment tool 3](#_Toc15890450)

[Supplementary Table S2. Characteristics of 63 eligible studies reporting overall survival at ≥2 years 4](#_Toc15890451)

[Supplementary Figure S1. Sensitivity analyses showing forest plots of 2-, 3-, and 5-year survival in studies with low risk of bias only stratified by recruitment period. 8](#_Toc15890452)

[Supplementary Figure S2. Sensitivity analyses showing forest plots of 2-, 3- and 5-year overall survival after removing studies using East Asian populations 9](#_Toc15890453)

[Supplementary Figure S3. Sensitivity analyses showing forest plots of 2-, 3- and 5- year overall survival after a change of recruitment period stratification to 2006 10](#_Toc15890454)

[Supplementary Figure S4. Sensitivity analyses showing forest plots of 2-, 3- and 5- year overall showing all non-overlapping populations within each stratified time period 11](#_Toc15890455)

[Supplementary references^1-34^ 13](#_Toc15890456)

# Appendix 1. Search Strategy

Search date: 15March2019

MEDLINE

1. (glioma or glioblastoma).tw
2. Population-based.tw or (population adj3 bas*).tw or (prefecture or nationwide or registry or register).tw
3. (Surviv* or mortality).tw
4. 1 and 2 and 3
5. Limit 4 to humans

Number of MEDLINE citations: 287

Embase

1. (glioma or glioblastoma).tw
2. Population-based.tw or (population adj3 bas*).tw or (prefecture or nationwide or registry or register).tw
3. (Surviv* or mortality).tw
4. (low adj3 grade).tw or p*ediatric.tw
5. (1 and 2 and 3) not 4
6. Limit 5 to humans

Number of Embase citations: 358

# Supplementary Table S1. Risk of bias assessment tool

|  | | | | | Y | | PY | PN | N | NI |
| --- | --- | --- | --- | --- | --- | --- | --- | --- | --- | --- |
| **Selection** | | | | |  | |  |  |  |  |
| Was selection of participants based on variables measured after the diagnosis of glioblastoma or start of exposure? | | | | |  | |  |  |  |  |
| Was selection of participants based on subclassification of glioblastoma? | | | | |  | |  |  |  |  |
| Were variables that influenced eligibility selection influenced by the outcome or a cause of the outcome? | | | | |  | |  |  |  |  |
| Were variables that influenced eligibility selection influenced the outcome? | | | | |  | |  |  |  |  |
| Do start of follow-up and start of exposure coincide for most participants? | | | | |  | |  |  |  |  |
| Were adjustment techniques used likely to correct for the presence of selection biases? | | | | |  | |  |  |  |  |
| Overall risk of bias (selection) | Low (1) | Moderate (2) | Serious (3) | Critical (4) | | No information (9) | | | | |
| **Exposure** | | | | |  | |  |  |  |  |
| Is exposure status well defined? | | | | |  | |  |  |  |  |
| Were exposure assessment methods robust? | | | | |  | |  |  |  |  |
| Could classification of exposure status have been affected by knowledge of the outcome or risk of the outcome? | | | | |  | |  |  |  |  |
| Is there concern that changes in exposure status occurred among participants? | | | | |  | |  |  |  |  |
| Did many participants switch to other exposures? | | | | |  | |  |  |  |  |
| Overall risk of bias (exposure) | Low (1) | Moderate (2) | Serious (3) | Critical (4) | | No information (9) | | | | |
| **Diagnosis** | | | | |  | |  |  |  |  |
| Were all eligible participants had histopathological diagnosis of glioblastoma? | | | | |  | |  |  |  |  |
| How likely would those non-histopathologically confirmed cases affect outcome? | | | | |  | |  |  |  |  |
| Overall risk of bias (diagnosis) | Low (1) | Moderate (2) | Serious (3) | Critical (4) | | No information (9) | | | | |
| **Confounding** | | | | |  | |  |  |  |  |
| Is there potential for confounding of the effect of exposure in this study? | | | | |  | |  |  |  |  |
| Use of appropriate analysis method for confounder adjustment? | | | | |  | |  |  |  |  |
| Overall risk of bias (confounding) | Low (1) | Moderate (2) | Serious (3) | Critical (4) | | No information (9) | | | | |
| **Missing** | | | | |  | |  |  |  |  |
| Were there missing outcome data? | | | | |  | |  |  |  |  |
| Were participants excluded due to missing data on exposure status? | | | | |  | |  |  |  |  |
| Were participants excluded due to missing data on other variables needed for the analysis? | | | | |  | |  |  |  |  |
| Are the proportion of participants and reasons for missing data similar across exposures? | | | | |  | |  |  |  |  |
| Where appropriate statistical methods used to account for missing data? | | | | |  | |  |  |  |  |
| Overall risk of bias (missing) | Low (1) | Moderate (2) | Serious (3) | Critical (4) | | No information (9) | | | | |
| **Outcome** | | | | |  | |  |  |  |  |
| Could the outcome measure have been influenced by knowledge of the exposure received? | | | | |  | |  |  |  |  |
| Were the methods of outcome assessment comparable across exposure groups? | | | | |  | |  |  |  |  |
| Were any systematic errors in measurement of the outcome unrelated to exposure received? | | | | |  | |  |  |  |  |
| Overall risk of bias (outcome) | Low (1) | Moderate (2) | Serious (3) | Critical (4) | | No information (9) | | | | |
|  | | | | |  | |  |  |  |  |
| OVERALL RISK OF BIAS | LOW | MODERATE | SERIOUS | CRITICAL | | NO INFOMRATION | | | | |

# Supplementary Table S2. Characteristics of 63 eligible studies reporting overall survival at ≥2 years

| Study | Country | Period | RoB^†^ | Patient selection | N | Age^‡^ | % female | % SR | % ChT | % RT | 2yr | 3yr | 5yr |
| --- | --- | --- | --- | --- | --- | --- | --- | --- | --- | --- | --- | --- | --- |
| Adams (2013) | US (SEER) | 1973-2009 | 2 | Excluded cases without tumour location, gliosarcoma, giant cell glioblastoma | 23329 | - | - | - | - | - | M | - | M |
| Al-Husseini (2018) | US (SEER) | 1973-2014 | 1 |  | 50243 | - | 43 | 43 | 47 | 71 | - | - | **M** |
| Arrigo (2012) | US (VA) | 1997-2006 | 3 | Supratentorial male cases only | 1219 | 63 | 0 | 74 | 31 | 66 | M | - | M |
| Asklund (2015) | Sweden | 1999-2912 | 1 |  | 1913 | - | - | - | - | - | **M** | **M** | **M** |
| Bauchet (2010) | France | 2004-2004 | 1 |  | 952 | 64 | 38 | 56 | 59 | 68 | **E** | - | - |
| Bergqvist (2018) | Sweden | 2001-2013 | 1 | 99% histologically confirmed cases | 968 | - | - | 43 | - | - | - | - | M |
| Bohn (2018) | US (SEER) | 2010-2014 | 3 | Excluded gliosarcoma and giant cell GBM, and cases with no information on race | 3473 | - | 42 | 84 | - | - | - | L | - |
| Brandes (2014) | Italy | 2001-2013 | 4 | Patient aged <70 years with KPS 30-100 treated with RT & TMZ | 139 | 59 | 38 | - | 100 | 100 | L | **L** | - |
| Brodbelt (2015) | UK | 2007-2011 | 1 |  | 10743 | 57 | 40 | 80 | 25 | - | **L** | - | **L** |
| Bruhn (2018) | Sweden (Jönköping) | 2001-2005 | 2 | Excluded secondary GBM and those still alive | 143 | - | 38 | 34 | - | - | **L** | - | - |
| Chan (2017) | Hong Kong | 2003-2005 | 4 | Excluded unstable neurology, KPS<70 post-op, or started on ChT other than TMZ | 68 | 52 | 35 | 75 | 38 | 62 | **M** | - | - |
| Chang (2005) | US (SEER) | 1988-2001 | 3 | Supratentorial GBM only. Excluded cases without marital status | 10987 | 64 | 43 | 74 | - | 74 | **E** | - | **E** |
| Chien (2015)* | US (Ohio) | 2007-2012 | 3 | Included 11% of all GBM diagnosed in Ohio. 10% with missing treatment details | 253 | - | - | - | - | - | L | - | - |
| Chien (2015)* | Taiwan | 2007-2012 | 1 |  | 908 | - | - | - | - | - | **L** | - | - |
| Dahlrot (2013) | Denmark (Southern) | 2005-2009 | 4 | Unexplained exclusion of 220 patients. 67 (19%) without histological diagnosis | 234 | 68 | - | - | - | - | L | - | - |
| Dubrow (2013) | US (VA) | 1997-2008 | 1 | Excluded 17 cases with insufficient treatment information | 1645 | - | 3 | 74 | 43 | 73 | **EL** | **EL** | **EL** |
| Eriksson (2019) | Sweden (Umeå) | 1995-2015 | 1 |  | 571 | - | 38 | - | - | - | E**L** | **EL** | **EL** |

GBM=glioblastoma multiforme; RoB=risk of bias SR=surgical resection; ChT=chemotherapy; RT=radiotherapy; KPS=Karnofsky Performance Score; TMZ=temozolomide; NOS=not otherwise specified; yr=years; %=percentage; VA=Veterans Health Administration; SEER=Surveillance, Epidemiology and End Results Program; OH=Ohio; CA=California; NC=North Carolina

*This is a single study reporting survival data on two separate population cohorts of GBM patients. ^†^Risk of bias relates to survival estimate reported in the study. Scores are based on assessment of patient selection, diagnostic certainty, handling of missing data and outcome measurement. Studies are categorized into 4 risk of bias groups: (1) low; (2) moderate; (3) serious; (4) critical. ^‡^Median or mean age of cohort in years depending on which was reported in the study. ^¥^Denotes availability of survival estimate and contribution to meta-analyses. E=survival estimate for period before 2005 (early); M=survival estimate for period across 2005 (middle); L=survival estimate for period during or after 2005 (late). A bold letter denotes contribution to meta-analyses.

| Study | Country | Period | RoB^†^ | Patient selection | N | Age^‡^ | % female | % SR | % ChT | % RT | 2yr | 3yr | 5yr |
| --- | --- | --- | --- | --- | --- | --- | --- | --- | --- | --- | --- | --- | --- |
| Fabbro-Peray (2018) | France | 2008-2008 | 1 |  | 2053 | 64 | 40 | 59 | 90 | 90 | **L** | - | **L** |
| Fekete (2016) | Sweden (Västra Götaland) | 2004-2008 | 1 |  | 229 | 60 | 41 | 84 | 49 | 58 | M | - | - |
| Frandsen (2018) | US (NCDB) | 2004-2013 | 2 | Excluded unknown chemo/radiotherapy status and non-conventional therapy. | 45858 | 61 | 34 | 25 | 58 | 62 | **M** | **M** | **M** |
| Fuentes-Raspall (2014) | Spain | 1994-2008 | 1 |  | 195 | 61 | 49 | - | - | - | - | **M** | M |
| Fuentes-Raspall (2017) | Spain | 1994-2013 | 1 |  | 463 | - | 43 | - | - | - | - | - | **M** |
| Gabriel (2014) | US (SEER) | 1973-2009 | 3 | Included adults older than 20 years, black racial demographic | 3272 | 0 | - | - | - | - | M | M | M |
| Gramatzki (2016) | Switzerland | 2005-2009 | 1 |  | 264 | 61 | 38 | 81 | 60 | 70 | **L** | **L** | - |
| Graus (2013) | Spain | 2008-2010 | 2 | Excluded those without follow-up. Public health system covers 24% of population | 834 | 62 | 39 | 66 | 61 | 72 | **L** | **L** | - |
| Gulati (2012) | Norway | 1998-2008 | 3 | 8% clinical diagnosis of glioblastoma without histological confirmation. Incomplete treatment data in 68%. | 2882 | 62 | - | 54 | 20 | 37 | **M** | - | - |
| Hansen (2018) | Denmark | 2009-2014 | 1 |  | 1362 | 66 | 39 | 74 | 59 | 76 | L | L | L |
| Ho (2014) | Netherlands | 1989-2010 | 3 | Included cases without histological confirmation. | 9402 | 61 | 40 | - | - | - | **M** | - | - |
| Iwamoto (2008) | US (SEER) | 1994-2002 | 4 | Medicare patients aged ≥65 years. Excluded cases with missing month of diagnosis, lacked part B coverage. | 5909 | - | 55 | 70 | 7 | 45 | E | **E** | E |
| Johnson (2012) | US (SEER) | 1998-2008 | 2 | Excluded patients who did not receive radiotherapy | 10022 | 59 | 59 | - | - | 100 | M | M | M |
| Johnson (2013) | US (SEER) | 2005-2010 | 2 | Excluded patients alive or without known survival time | 5607 | 62 | 41 | 79 | 48 | 73 | EML | - | - |
| Johnson (2018) | US (SEER) | 2006-2012 | 1 | Excluded patients diagnosed in 2006 when bevacizumab was approved | 12873 | - | 41 | 61 | 51 | 75 | L | **L** | - |

GBM=glioblastoma multiforme; RoB=risk of bias SR=surgical resection; ChT=chemotherapy; RT=radiotherapy; KPS=Karnofsky Performance Score; TMZ=temozolomide; NOS=not otherwise specified; yr=years; %=percentage; VA=Veterans Health Administration; SEER=Surveillance, Epidemiology and End Results Program; OH=Ohio; CA=California; NC=North Carolina

*This is a single study reporting survival data on two separate population cohorts of GBM patients. ^†^Risk of bias relates to survival estimate reported in the study. Scores are based on assessment of patient selection, diagnostic certainty, handling of missing data and outcome measurement. Studies are categorized into 4 risk of bias groups: (1) low; (2) moderate; (3) serious; (4) critical. ^‡^Median or mean age of cohort in years depending on which was reported in the study. ^¥^Denotes availability of survival estimate and contribution to meta-analyses. E=survival estimate for period before 2005 (early); M=survival estimate for period across 2005 (middle); L=survival estimate for period during or after 2005 (late). A bold letter denotes contribution to meta-analyses.

| Study | Country | Period | RoB^†^ | Patient selection | N | Age^‡^ | % female | % SR | % ChT | % RT | 2yr | 3yr | 5yr |
| --- | --- | --- | --- | --- | --- | --- | --- | --- | --- | --- | --- | --- | --- |
| Jung (2012) | Korea | 1999-2007 | 1 |  | 2751 | - | - | - | - | - | **EL** | **E** | **E** |
| Kim (2019 | US (SEER) | 2005-2014 | 4 | Included patients aged ≥20 years who had chemoradiotherapy with follow up details | 9911 | - | 40 | 77 | 100 | 100 | M | M | M |
| Kita (2009) | Switzerland | 1980-1994 | 4 | 21% clinical diagnosis without histological confirmation | 715 | 61 | 40 | 54 | - | 43 | E | - | - |
| Mak (2017) | US (NCDB) | 1998-2011 | 4 | Aged ≥70 years and with radiation dose fractionation details | 4598 | 75 | 45 | 80 | 77 | 100 | M | M | - |
| Mathiesen (2011) | Sweden | 1996-2001 | 1 |  | 1110 | - | - | - | - | - | **E** | - | - |
| McLendon (2011) | US (NC) | 1976-1996 | 1 | Excluded patients without 5-year follow up data | 766 | - | - | - | - | - | - | - | E |
| Morgan (2017) | Canada | 2006-2012 | 1 | Excluded those had initial therapy or most treatment administered out of province | 138 | 61 | 39 | 83 | 65 | 87 | **L** | - | - |
| Narita (2015) | Japan | 1989-2008 | 1 | Patients who had been under treatment for ≥5 years | 1489 | - | - | - | - | - | - | - | **M** |
| Nava (2014) | Italy | 1997-2010 | 1 | Excluded secondary GBM | 1254 | - | 36 | 91 | - | - | **EM** | **EM** | **EM** |
| Nobusawa (2009) | Switzerland | 1980-1994 | 3 | 19% without histology. Survival available from cases treated with resection and radiotherapy | 407 | 60 | 41 | - | - | - | E | E | - |
| Nuno (2014) | US (SEER) | 1991-2007 | 4 | Aged ≥65 years; excluded those died within 1 month | 2774 | 73 | 48 | 79 | - | - | M | - | - |
| Ohgaki (2004) | Switzerland | 1980-1994 | 3 | Autopsy cases included; 20% cases without histopathological diagnosis | 715 | 61 | - | - | - | - | **E** | **E** | - |
| Ortega (2014) | US (VA) | 1988-2011 | 1 |  | 67509 | 61 | 43 | 57 | 63 | 77 | **M** | - | - |
| Ostrom (2018) | US (SEER) | 2000-2014 | 3 | Survival data taken from survival curves of individuals who received resection | 11923 | - | - | - | - | - | M | - | - |
| Pan (2015) | US (SEER) | 2000-2009 | 1 |  | 14675 | 61 | 41 | 75 | - | 75 | M | - | - |
| Pitz (2012) | Canada | 2002-2009 | 4 | Patients who started chemoradiotherapy only | 116 | 58 | 41 | - | - | - | M | M | M |
| Pretanvil (2017) | US (CA) | 2005-2010 | 1 |  | 2670 | - | - | 72 | 63 | 71 | M | M | M |

GBM=glioblastoma multiforme; RoB=risk of bias SR=surgical resection; ChT=chemotherapy; RT=radiotherapy; KPS=Karnofsky Performance Score; TMZ=temozolomide; NOS=not otherwise specified; yr=years; %=percentage; VA=Veterans Health Administration; SEER=Surveillance, Epidemiology and End Results Program; OH=Ohio; CA=California; NC=North Carolina

*This is a single study reporting survival data on two separate population cohorts of GBM patients. ^†^Risk of bias relates to survival estimate reported in the study. Scores are based on assessment of patient selection, diagnostic certainty, handling of missing data and outcome measurement. Studies are categorized into 4 risk of bias groups: (1) low; (2) moderate; (3) serious; (4) critical. ^‡^Median or mean age of cohort in years depending on which was reported in the study. ^¥^Denotes availability of survival estimate and contribution to meta-analyses. E=survival estimate for period before 2005 (early); M=survival estimate for period across 2005 (middle); L=survival estimate for period during or after 2005 (late). A bold letter denotes contribution to meta-analyses.

| Study | Country | Period | RoB^†^ | Patient selection | N | Age^‡^ | % female | % SR | % ChT | % RT | 2yr^¥^ | 3yr^¥^ | 5yr^¥^ |
| --- | --- | --- | --- | --- | --- | --- | --- | --- | --- | --- | --- | --- | --- |
| Rasmussen (2017) | Denmark | 2009-2014 | 1 |  | 1364 | 64 | 61 | - | - | - | **L** | **L** | **L** |
| Rong (2016) | US (SEER) | 2007-2012 | 4 | Excluded 29567 patients with unknown insurance status. “Large proportion” without histological confirmation | 13665 | 63 | 42 | 76 | - | 60 | **L** | - | **L** |
| Ronning (2012) | Norway | 2000-2007 | 1 |  | 1157 | 63 | 40 | - | - | - | EM | - | - |
| Rosenthal (2006) | Australia | 1998-2000 | 2 | Included patients who returned questionnaire | 473 | - | - | - | - | - | **E** | **E** | **E** |
| Rusthoven (2014) | US (SEER) | 1998-2007 | 3 | Included patients who had upfront surgical resection; excluded “surgery NOS” cases | 12115 | 73 | 41 | 100 | - | 82 | M | - | - |
| Rusthoven (2016) | US (SEER) | 2005-2011 | 4 | Patients aged ≥65 years with complete data | 16717 | - | 47 | 68 | 57 | 61 | M | M | - |
| Salmaggi (2008) | Italy (Lombardy) | 2005-2005 | 3 |  | 349 | 60 | 36 | 70 | 100 | 89 | **L** | - | - |
| Seliger (2019) | Germany | 1998-2013 | 2 | Excluded 10% without follow up; no information regarding these cases | 862 | - | - | - | - | - | M | M | M |
| Seliger (2018) | Germany | 1998-2013 | 2 | Excluded 10% without follow up; no information regarding these cases | 862 | - | - | - | - | - | **M** | **M** | **M** |
| Shah (2016) | US (SEER) | 1994-2010 | 4 | Aged ≥70 years; excluded cases with unknown marital status, radiation status, tumor location | 6039 | - | 48 | 71 | - | 61 | EL | EL | - |
| Tian (2018) | US (SEER) | 2000-2008 | 4 | Aged 18-70 who underwent surgical treatment; excluded those with incomplete data | 6586 | - | 39 | 100 | - | - | - | M | M |
| Walker (2013) | US (SEER) | 1973-2006 | 4 | Supratentorial GBM only; excluded patients who died within 1 month | 9103 | 63 | 42 | 78 | - | 77 | M | - | - |
| Wrensch (2006) | US (SEER) | 1991-1999 | 1 | Age ≥20 | 517 | - | - | - | - | - | E | - | - |
| Xu (2017) | US (SEER) | 2004-2013 | 1 |  | 24262 | 62 | 43 | 72 | - | 70 | - | **M** | M |
| Yuan (2016) | Canada | 1992-2008 | 4 | 25% cases without histopathological diagnosis | 14120 | - | - | - | - | - | **M** | **M** | **M** |

GBM=glioblastoma multiforme; RoB=risk of bias SR=surgical resection; ChT=chemotherapy; RT=radiotherapy; KPS=Karnofsky Performance Score; TMZ=temozolomide; NOS=not otherwise specified; yr=years; %=percentage; VA=Veterans Health Administration; SEER=Surveillance, Epidemiology and End Results Program; OH=Ohio; CA=California; NC=North Carolina

*This is a single study reporting survival data on two separate population cohorts of GBM patients. ^†^Risk of bias relates to survival estimate reported in the study. Scores are based on assessment of patient selection, diagnostic certainty, handling of missing data and outcome measurement. Studies are categorized into 4 risk of bias groups: (1) low; (2) moderate; (3) serious; (4) critical. ^‡^Median or mean age of cohort in years depending on which was reported in the study. ^¥^Denotes availability of survival estimate and contribution to meta-analyses. E=survival estimate for period before 2005 (early); M=survival estimate for period across 2005 (middle); L=survival estimate for period during or after 2005 (late). A bold letter denotes contribution to meta-analyses.

Supplementary Figure S1. Sensitivity analyses showing forest plots of 2-, 3-, and 5-year survival in studies with low risk of bias only stratified by recruitment period.


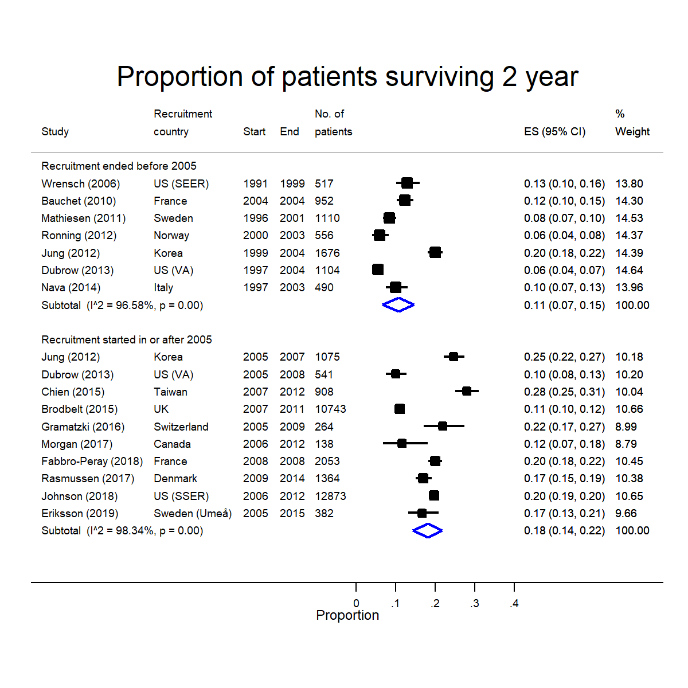


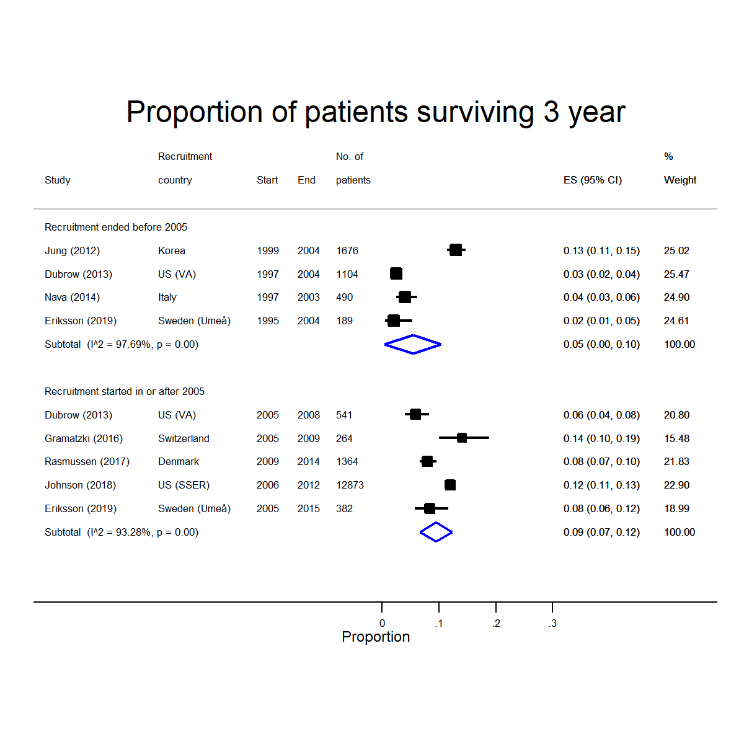

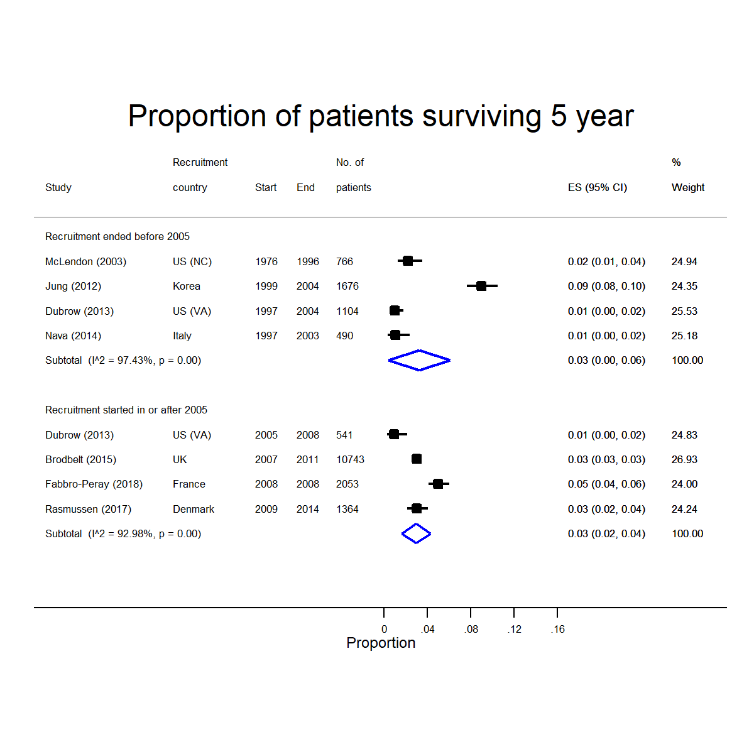


# **
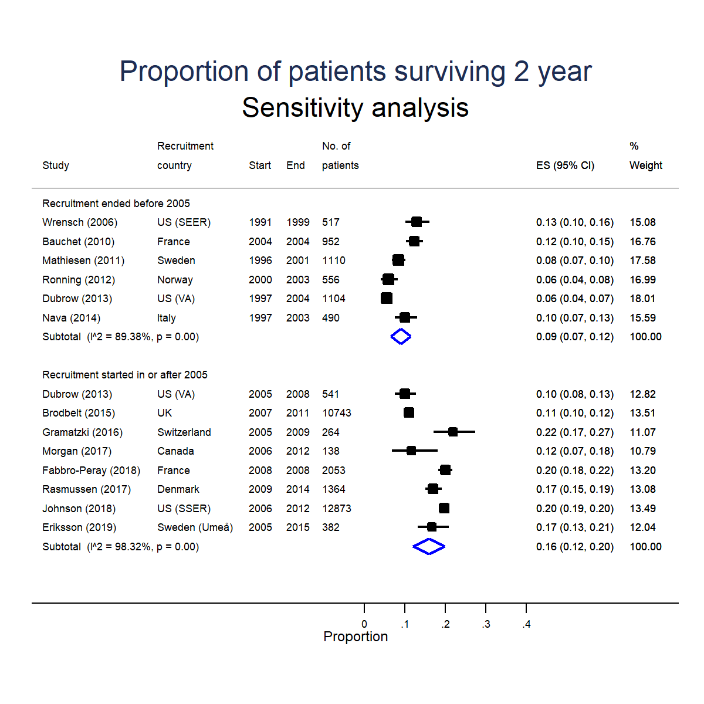
Supplementary Figure S2**. **Sensitivity analyses showing** forest plots of 2-, 3- and 5-year overall survival after removing studies using East Asian populations


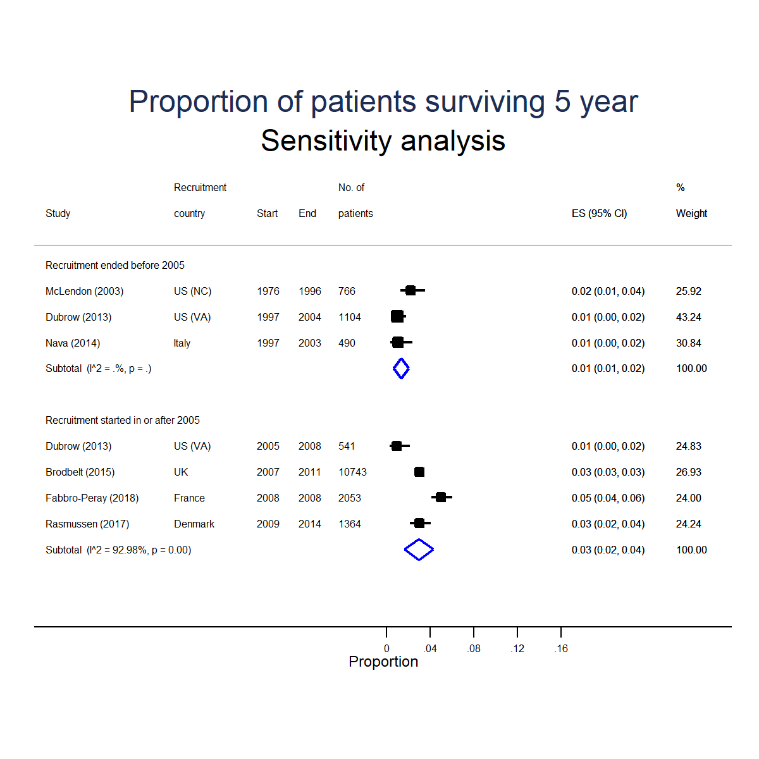

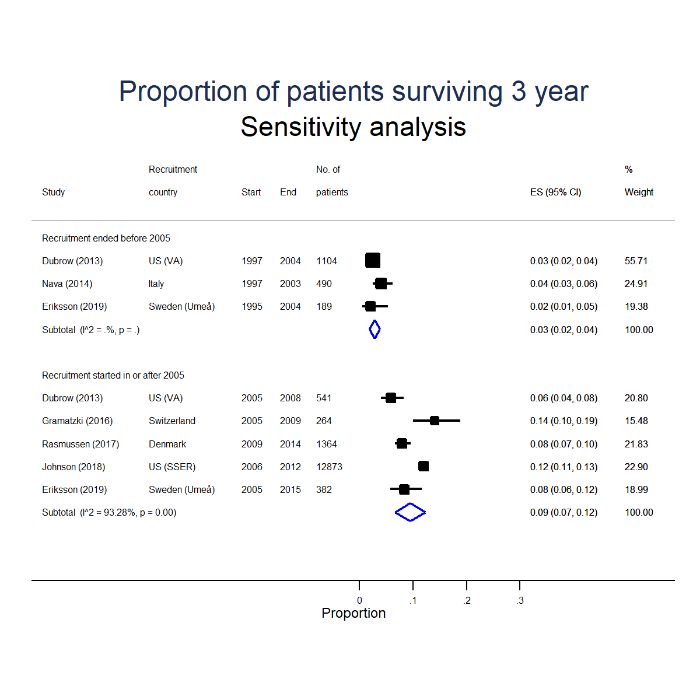


#
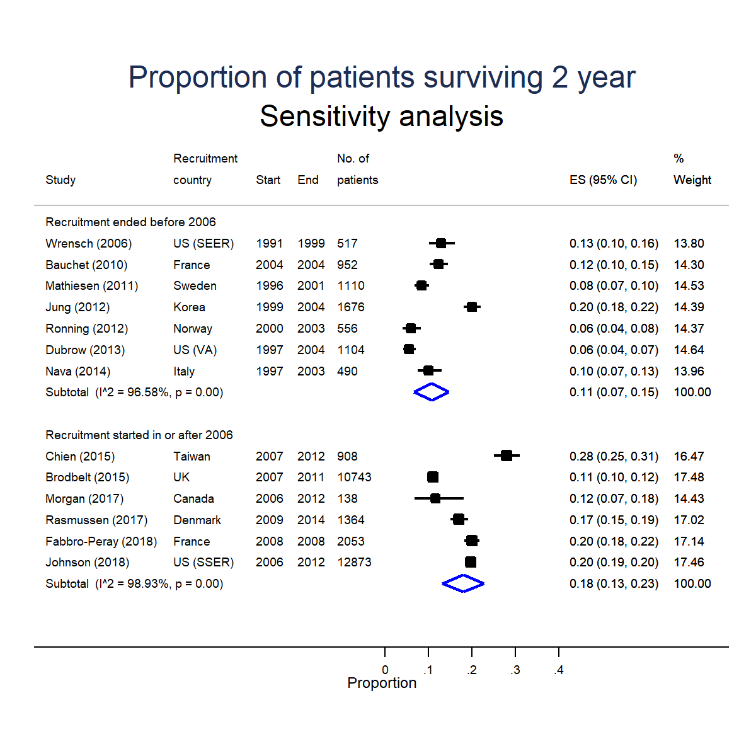
**Supplementary Figure** S3. Sensitivity analyses showing forest plots of 2-, 3- and 5- year overall survival after a change of recruitment period stratification to 2006


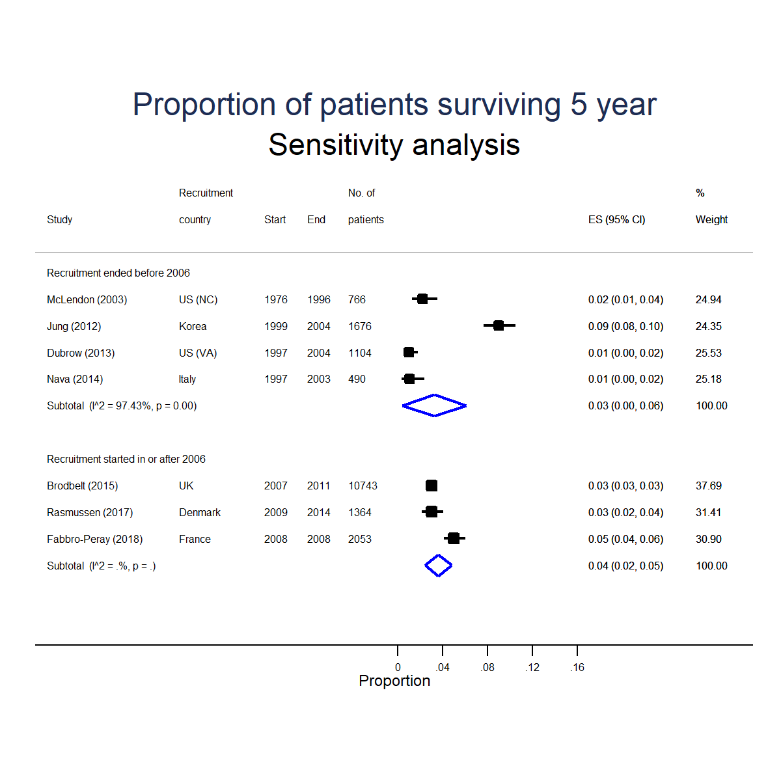

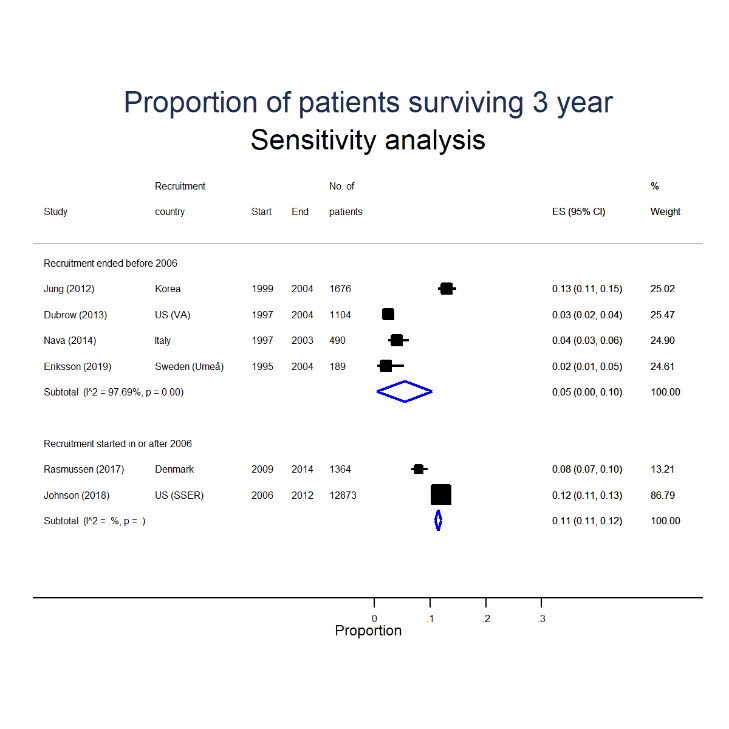


# **Supplementary Figure** S4. Sensitivity analyses showing forest plots of 2-, 3- and 5- year overall showing all non-overlapping populations within each stratified time period


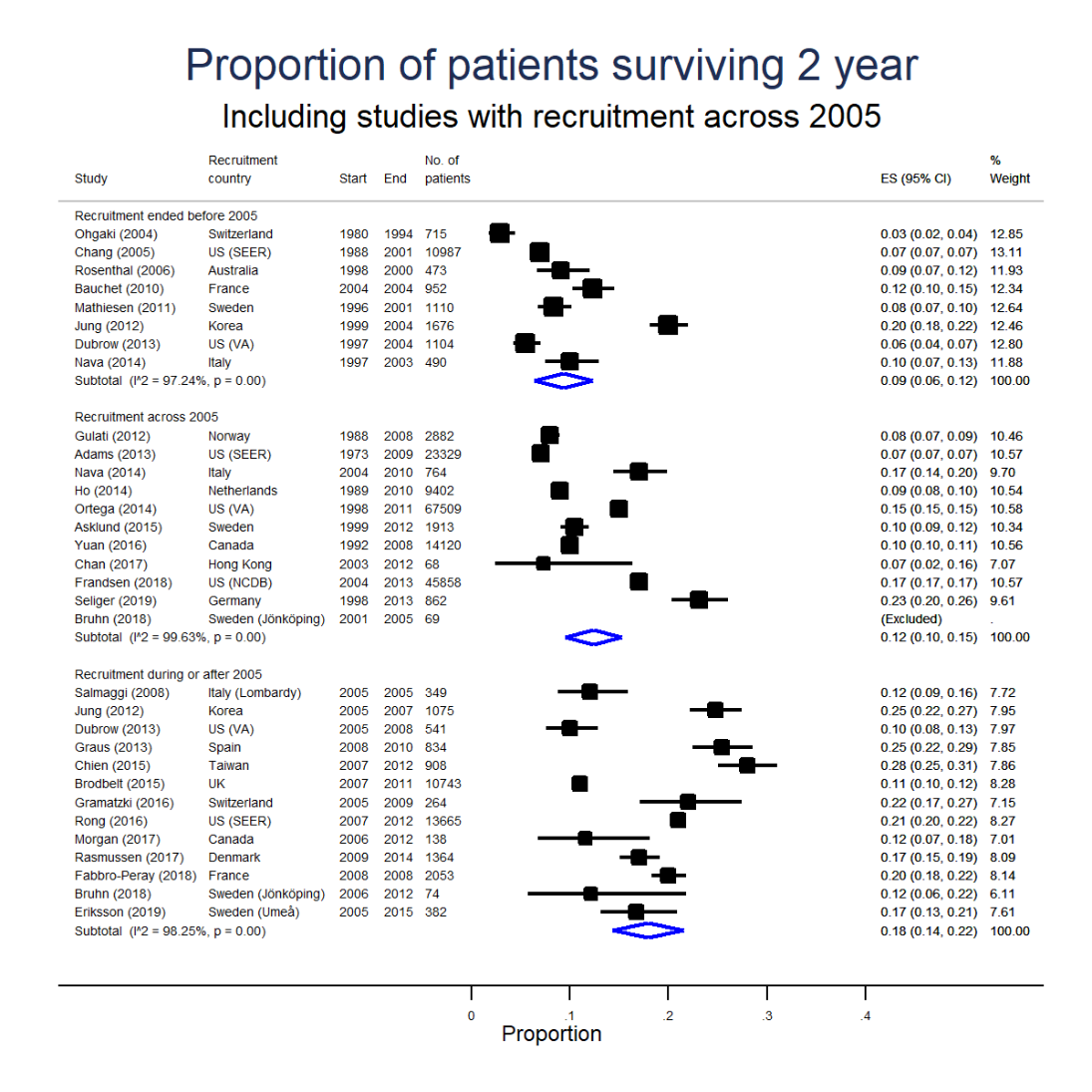


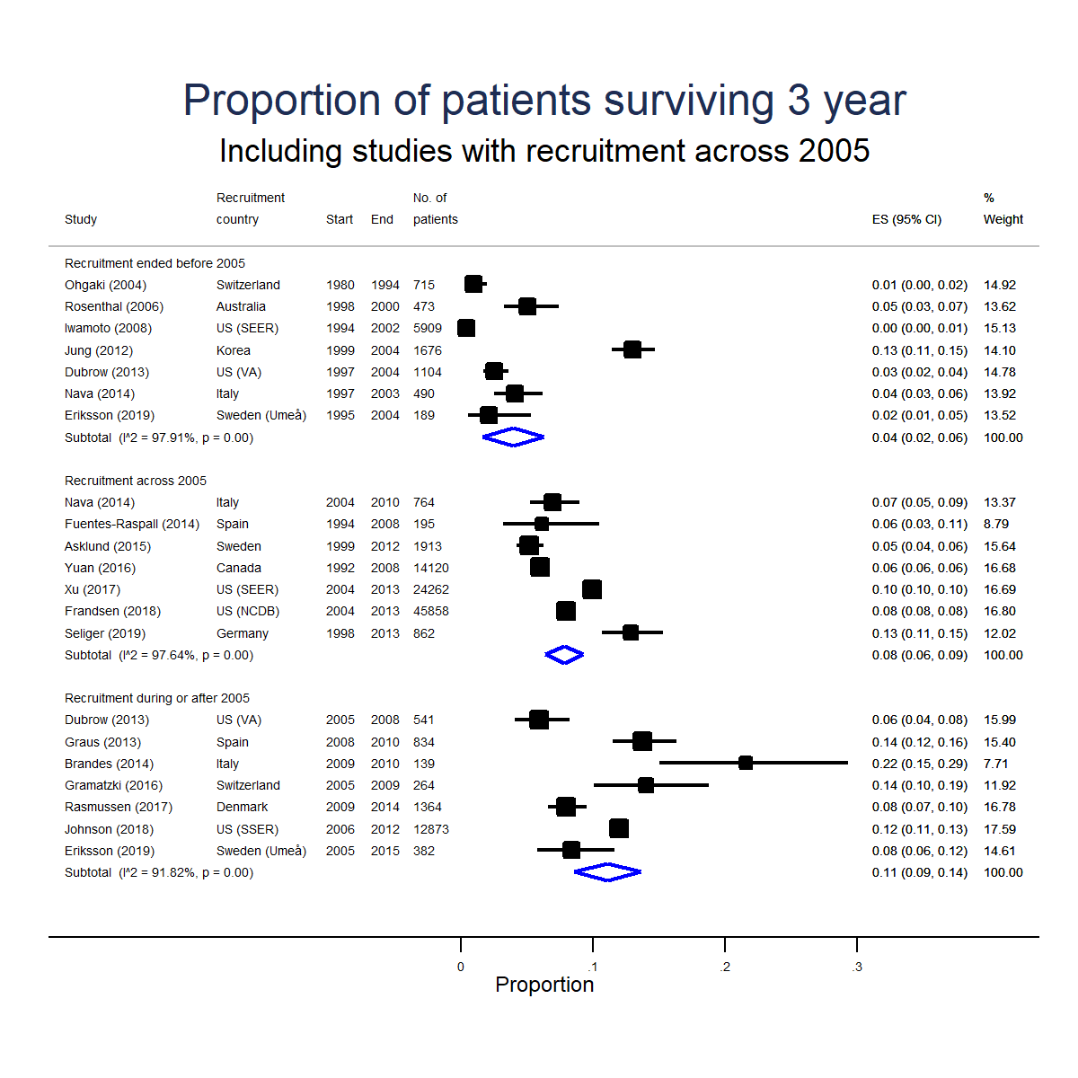


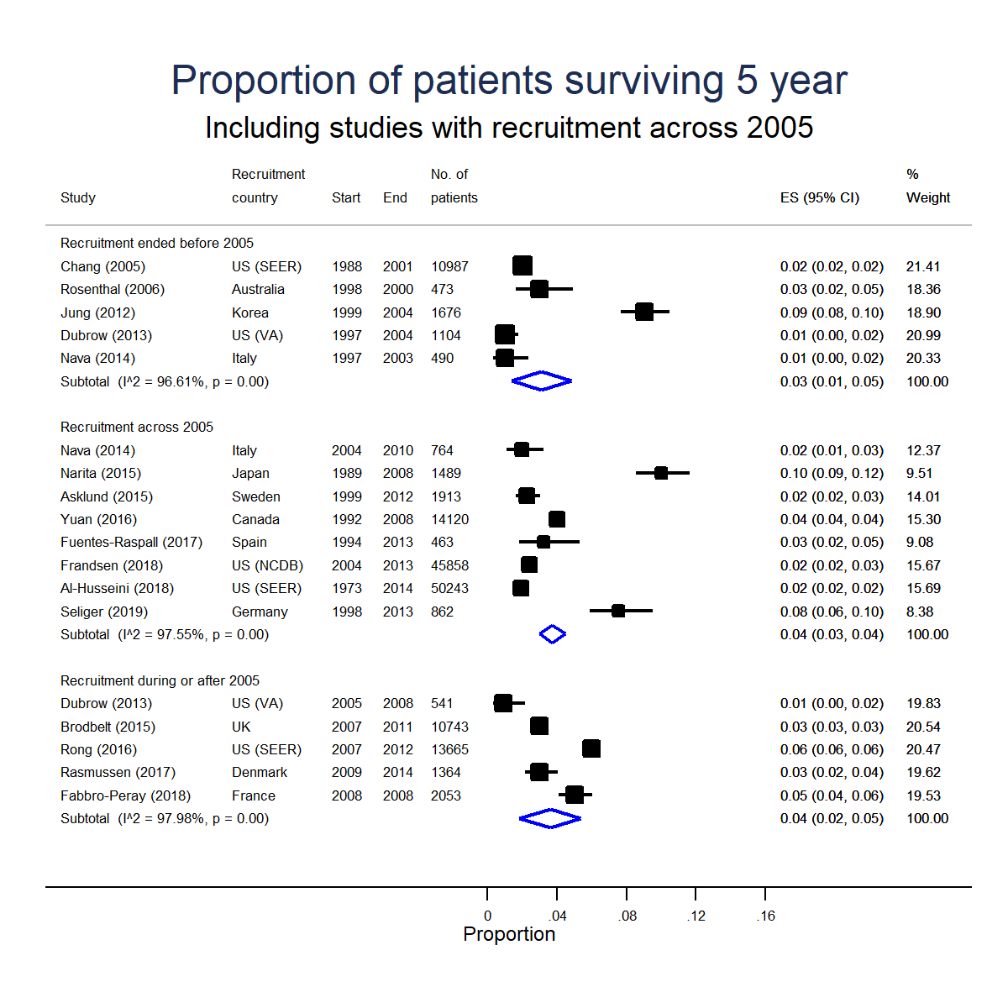


Note that there are overlapping patients between the middle and the other groups.

# Supplementary references^1-34^

1. Adams H, Chaichana KL, Avendano J, et al. Adult cerebellar glioblastoma: understanding survival and prognostic factors using a population-based database from 1973 to 2009. *World neurosurgery* 2013;80(6):e237-43. doi: https://dx.doi.org/10.1016/j.wneu.2013.02.010

2. Al-Husseini MJ, Saad AM, El-Shewy KM, et al. Prior malignancy impact on survival outcomes of glioblastoma multiforme; population-based study. *International Journal of Neuroscience* 2018 doi: http://dx.doi.org/10.1080/00207454.2018.1538989

3. Arrigo RT, Boakye M, Skirboll SL. Patterns of care and survival for glioblastoma patients in the Veterans population. *Journal of neuro-oncology* 2012;106(3):627-35. doi: https://dx.doi.org/10.1007/s11060-011-0702-6

4. Asklund T, Malmstrom A, Bergqvist M, et al. Brain tumors in Sweden: data from a population-based registry 1999-2012. *Acta oncologica (Stockholm, Sweden)* 2015;54(3):377-84. doi: https://dx.doi.org/10.3109/0284186X.2014.975369

5. Bergqvist J, Iderberg H, Mesterton J, et al. The effects of clinical and sociodemographic factors on survival, resource use and lead times in patients with high-grade gliomas: a population-based register study. *Journal of neuro-oncology* 2018;139(3):599-608. doi: https://dx.doi.org/10.1007/s11060-018-2899-0

6. Bohn A, Braley A, Rodriguez de la Vega P, et al. The association between race and survival in glioblastoma patients in the US: A retrospective cohort study. *PLoS One* 2018;13(6):e0198581. doi: 10.1371/journal.pone.0198581

7. Chan DT, Hsieh SY, Lau CK, et al. Ten-year review of survival and management of malignant glioma in Hong Kong. *Hong Kong medical journal = Xianggang yi xue za zhi* 2017;23(2):134-9. doi: https://dx.doi.org/10.12809/hkmj164879

8. Dahlrot RH, Kristensen BW, Hjelmborg J, et al. A population-based study of high-grade gliomas and mutated isocitrate dehydrogenase 1. *Int J Clin Exp Pathol* 2013;6(1):31-40.

9. Fekete B, Werlenius K, Orndal C, et al. Prognostic factors for glioblastoma patients--a clinical population-based study. *Acta neurologica Scandinavica* 2016;133(6):434-41. doi: https://dx.doi.org/10.1111/ane.12481

10. Frandsen J, Orton A, Jensen R, et al. Patterns of care and outcomes in gliosarcoma: An analysis of the National Cancer Database. *Journal of Neurosurgery* 2018;128(4):1133-38. doi: http://dx.doi.org/10.3171/2016.12.JNS162291

11. Fuentes-Raspall R, Puig-Vives M, Guerra-Prio S, et al. Population-based survival analyses of central nervous system tumors from 1994 to 2008. An up-dated study in the temozolomide-era. *Cancer epidemiology* 2014;38(3):244-7. doi: https://dx.doi.org/10.1016/j.canep.2014.03.014

12. Fuentes-Raspall R, Solans M, Roca-Barcelo A, et al. Descriptive epidemiology of primary malignant and non-malignant central nervous tumors in Spain: Results from the Girona Cancer Registry (1994-2013). *Cancer epidemiology* 2017;50(Pt A):1-8. doi: https://dx.doi.org/10.1016/j.canep.2017.07.005

13. Gabriel A, Batey J, Capogreco J, et al. Adult brain cancer in the U.S. black population: a Surveillance, Epidemiology, and End Results (SEER) analysis of incidence, survival, and trends. *Medical science monitor : international medical journal of experimental and clinical research* 2014;20:1510-7. doi: https://dx.doi.org/10.12659/MSM.890762

14. Gulati S, Jakola AS, Johannesen TB, et al. Survival and treatment patterns of glioblastoma in the elderly: a population-based study. *World neurosurgery* 2012;78(5):518-26. doi: https://dx.doi.org/10.1016/j.wneu.2011.12.008

15. Hansen S, Rasmussen BK, Laursen RJ, et al. Treatment and survival of glioblastoma patients in Denmark: The Danish Neuro-Oncology Registry 2009-2014. *Journal of Neuro-Oncology* 2018;139(2):479-89. doi: http://dx.doi.org/10.1007/s11060-018-2892-7

16. Ho VKY, Reijneveld JC, Enting RH, et al. Changing incidence and improved survival of gliomas. *European journal of cancer (Oxford, England : 1990)* 2014;50(13):2309-18. doi: https://dx.doi.org/10.1016/j.ejca.2014.05.019

17. Johnson DR, Leeper HE, Uhm JH. Glioblastoma survival in the United States improved after Food and Drug Administration approval of bevacizumab: a population-based analysis. *Cancer* 2013;119(19):3489-95. doi: https://dx.doi.org/10.1002/cncr.28259

18. Johnson DR, Ma DJ, Buckner JC, et al. Conditional probability of long-term survival in glioblastoma: a population-based analysis. *Cancer* 2012;118(22):5608-13. doi: https://dx.doi.org/10.1002/cncr.27590

19. Kim YJ, Lee DJ, Park CK, et al. Optimal extent of resection for glioblastoma according to site, extension, and size: a population-based study in the temozolomide era. *Neurosurgical Review* 2019 doi: http://dx.doi.org/10.1007/s10143-018-01071-3

20. Kita D, Ciernik IF, Vaccarella S, et al. Age as a predictive factor in glioblastomas: population-based study. *Neuroepidemiology* 2009;33(1):17-22. doi: https://dx.doi.org/10.1159/000210017

21. McLendon RE, Halperin EC. Is the long-term survival of patients with intracranial glioblastoma multiforme overstated? *Cancer* 2003;98(8):1745-8.

22. Narita Y, Shibui S, Committee of Brain Tumor Registry of Japan Supported by the Japan Neurosurgical S. Trends and outcomes in the treatment of gliomas based on data during 2001-2004 from the Brain Tumor Registry of Japan. *Neurologia medico-chirurgica* 2015;55(4):286-95. doi: https://dx.doi.org/10.2176/nmc.ra.2014-0348

23. Nobusawa S, Watanabe T, Kleihues P, et al. IDH1 mutations as molecular signature and predictive factor of secondary glioblastomas. *Clinical cancer research : an official journal of the American Association for Cancer Research* 2009;15(19):6002-7. doi: https://dx.doi.org/10.1158/1078-0432.CCR-09-0715

24. Ortega A, Nuno M, Walia S, et al. Treatment and survival of patients harboring histological variants of glioblastoma. *Journal of clinical neuroscience : official journal of the Neurosurgical Society of Australasia* 2014;21(10):1709-13. doi: https://dx.doi.org/10.1016/j.jocn.2014.05.003

25. Pan IW, Ferguson SD, Lam S. Patient and treatment factors associated with survival among adult glioblastoma patients: A USA population-based study from 2000-2010. *Journal of clinical neuroscience : official journal of the Neurosurgical Society of Australasia* 2015;22(10):1575-81. doi: https://dx.doi.org/10.1016/j.jocn.2015.03.032

26. Pitz MW, Lipson M, Hosseini B, et al. Extended adjuvant temozolomide with cis-retinoic acid for adult glioblastoma. *Current Oncology* 2012;19(6):308-14. doi: http://dx.doi.org/10.3747/co.19.1151

27. Pretanvil J-A, Salinas IQ, Piccioni DE. Glioblastoma in the elderly: treatment patterns and survival. *CNS oncology* 2017;6(1):19-28. doi: https://dx.doi.org/10.2217/cns-2016-0023

28. Ronning PA, Helseth E, Meling TR, et al. A population-based study on the effect of temozolomide in the treatment of glioblastoma multiforme. *Neuro-oncology* 2012;14(9):1178-84. doi: https://dx.doi.org/10.1093/neuonc/nos153

29. Seliger C, Luber C, Gerken M, et al. Use of metformin and survival of patients with high-grade glioma. *International Journal of Cancer* 2019;144(2):273-80. doi: http://dx.doi.org/10.1002/ijc.31783

30. Seliger C, Schaertl J, Gerken M, et al. Use of statins or NSAIDs and survival of patients with high-grade glioma. *PLoS ONE* 2018;13(12):e0207858. doi: http://dx.doi.org/10.1371/journal.pone.0207858

31. Tian M, Ma W, Chen Y, et al. Impact of gender on the survival of patients with glioblastoma. *Bioscience Reports* 2018;38(6):BSR20180752. doi: http://dx.doi.org/10.1042/BSR20180752

32. Walker GV, Li J, Mahajan A, et al. Decreasing radiation therapy utilization in adult patients with glioblastoma multiforme: a population-based analysis. *Cancer* 2012;118(18):4538-44. doi: https://dx.doi.org/10.1002/cncr.27439

33. Wrensch M, Rice T, Miike R, et al. Diagnostic, treatment, and demographic factors influencing survival in a population-based study of adult glioma patients in the San Francisco Bay Area. *Neuro-oncology* 2006;8(1):12-26.

34. Xu H, Chen J, Xu H, et al. Geographic variations in the incidence of glioblastoma and prognostic factors predictive of overall survival in US adults from 2004-2013. *Frontiers in Aging Neuroscience* 2017;9(NOV):352. doi: http://dx.doi.org/10.3389/fnagi.2017.00352
